# Accelerated search for materials with targeted properties by adaptive design

**DOI:** 10.1038/ncomms11241

**Published:** 2016-04-15

**Authors:** Dezhen Xue, Prasanna V. Balachandran, John Hogden, James Theiler, Deqing Xue, Turab Lookman

**Affiliations:** 1 Theoretical Division, Los Alamos National Laboratory, MS-B262, Los Alamos, New Mexico 87545, USA; 2 State Key Laboratory for Mechanical Behavior of Materials, Xi'an Jiaotong University, Xi'an 710049, China; 3 Computer and Computational Sciences, Los Alamos National Laboratory, Los Alamos, New Mexico 87545, USA; 4 Intelligence and Space Research, Los Alamos National Laboratory, Los Alamos, New Mexico 87545, USA

## Abstract

Finding new materials with targeted properties has traditionally been guided by intuition, and trial and error. With increasing chemical complexity, the combinatorial possibilities are too large for an Edisonian approach to be practical. Here we show how an adaptive design strategy, tightly coupled with experiments, can accelerate the discovery process by sequentially identifying the next experiments or calculations, to effectively navigate the complex search space. Our strategy uses inference and global optimization to balance the trade-off between exploitation and exploration of the search space. We demonstrate this by finding very low thermal hysteresis (Δ*T*) NiTi-based shape memory alloys, with Ti_50.0_Ni_46.7_Cu_0.8_Fe_2.3_Pd_0.2_ possessing the smallest Δ*T* (1.84 K). We synthesize and characterize 36 predicted compositions (9 feedback loops) from a potential space of ∼800,000 compositions. Of these, 14 had smaller Δ*T* than any of the 22 in the original data set.

## Abstract (web summary)

Design of materials with targeted properties requires innovative approaches to guide researchers through complex search space. Here, the authors report an adaptive design strategy, using inference and global optimization methods, which can find shape memory alloys with very low thermal hysteresis.

## Graphical abstract

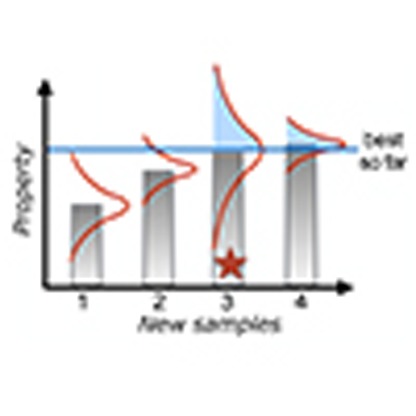

There has been much recent interest in accelerating materials discovery^1^. High-throughput calculations^2^,^3^ and combinatorial experiments^4^ have been the approaches of choice to narrow the search space. However, the interplay of structural, chemical and microstructural degrees of freedom introduces enormous complexity, especially if defects, solid solutions, non-stoichiometry and multicomponent compounds are involved, which the current state-of-the-art tools are not yet designed to handle. Statistical inference and machine learning algorithms have also been recently applied to materials design problems^5^,^6^. The emphasis has largely been on feature or descriptor selection or the use of regression tools, such as least squares, to predict properties. The regression studies have been hampered by small data sets, large model or prediction uncertainties and extrapolation to a vast unexplored chemical space with little or no experimental feedback to validate the predictions. Thus, they are prone to be suboptimal^7^, because they only exploit the model outcome and are liable to be confined to local minima, without sampling points in the search space where the uncertainties are largest. Hence, an approach is needed, which can adaptively guide the next experiments. That is, an adaptive procedure that makes optimal choices of materials to test next by balancing the merits of searching for materials likely to have the best property or where there may be fewer sampling points and greater uncertainty, but which may improve the quality of the regressor in the long run. Adaptive design has been successfully applied in areas spanning computer science^8^, operations research^7^ and cancer genomics^9^. The novelty of this approach is that it provides a robust, guided basis for the selection of the next material for experimental measurements by using uncertainties and maximizing the ‘expected improvement' from the best-so-far material in an iterative loop with feedback from experiments. It balances the goal of searching materials likely to have the best property (exploitation) with the need to explore parts of the search space with fewer sampling points and greater uncertainty.

Our goal here is to find new multicomponent NiTi-based shape memory alloys (SMAs) with the targeted property of very low thermal hysteresis (Δ*T*). The functionalities of SMAs, including shape memory effect and superelasticity, arise from the reversible martensitic transformation between high-temperature austenite and low-temperature martensite phases. Heating and cooling across the martensitic transformation temperature results in hysteresis (Δ*T*) as the transformation temperatures do not coincide, giving rise to fatigue. In Ni_50_Ti_50_ alloy, one of the best known SMA materials, it has been shown that 60 heating and cooling cycles results in a shift in the transformation temperature of 25 K, indicating poor resistance to fatigue^10^. Therefore, minimizing Δ*T* is crucial for realizing NiTi-based SMA applications^11^. A common strategy is to chemically modify Ni_50_Ti_50_ by substitutions at the Ni site. An obstacle for developing low ΔT SMAs is the large search space, because a vast majority of transition metals can be alloyed with Ni_50_Ti_50_. We, however, constrain the problem to the Ni_50−*x*−*y*−*z*_Ti_50_Cu_*x*_Fe_*y*_Pd_*z*_ family, where recent experimental results have shown promise^10^,^11^. This family of alloys can undergo a cubic to rhombhohedral (B2→R) or cubic to orthorhombic, monoclinic (B2→B19, B2→B19′) transformation (Supplementary Fig. 1) and both types have potential niche applications in industry^12^,^13^. The concentrations *x*, *y* and *z* dictate which transformations will be realized and the objective of our adaptive design here is to find *x*, *y*, *z* leading to the lowest Δ*T*, that is, the global optimum. It is a rich problem in that there are several transformations and our algorithm has to navigate the search space.

Recent efforts to find low dissipation SMAs have included the use of physical principles based on the crystallographic theory of martensite^10^. It has been pointed out that the middle eigenvalue (*λ*_2_) of the distortion matrix that deforms unstressed austenite into the deformed martensite structure should be close to 1, to minimize the effects of hysteresis^14^. The *λ*_2_=1 condition ensures that strain compatibility is exactly satisfied between austenite and martensite. This rule of thumb has been used to find ternary^15^ and quaternary alloys^10^ with small hysteresis based on high-throughput experiments. However, calculation of *λ*_2_ requires a priori knowledge of crystal symmetry and lattice parameters, typically obtained from diffraction measurements after experimental synthesis and, therefore, the approach has limited predictive power (especially in multicomponent alloys where the changes in lattice parameter as a function of composition are not known, as in this work). Furthermore, *λ*_2_=1 is an elastic condition and it is possible for two or more alloys with *λ*_2_ close to 1 to possess quite different Δ*T* (see Supplementary Fig. 2 and Supplementary Note 1), in which case thermal hysteresis would also be influenced by thermodynamics (Supplementary Fig. 3). Therefore, *λ*_2_=1 is only a necessary and not sufficient condition for finding low Δ*T* alloys. What is desired is a one-to-one direct mapping between alloy compositions and Δ*T*, which we establish using inference methods.

Our objective is to find alloys undergoing R, B19 or B19′ transformations, yet efficiently find the global minimum for Δ*T*. Even in our constrained pseudo-quaternary composition space, there are *N*=797,504 potential alloys. Such a vast space is difficult to explore with high-throughput experiments or *ab initio* calculations. However, our design loop is able to discover Ti_50.0_Ni_46.7_Cu_0.8_Fe_2.3_Pd_0.2_ with a low Δ*T* of 1.84 K in the sixth out of nine iterations of our loop. In all, 14 alloys are found with Δ*T* <3.15 K, the best value in our original experimental data. Our design framework thus accelerates the process of finding materials with desired properties offering the opportunity to significantly reduce the number of costly and time-consuming experiments.

## Results

### Design loop

**Figure 1:**
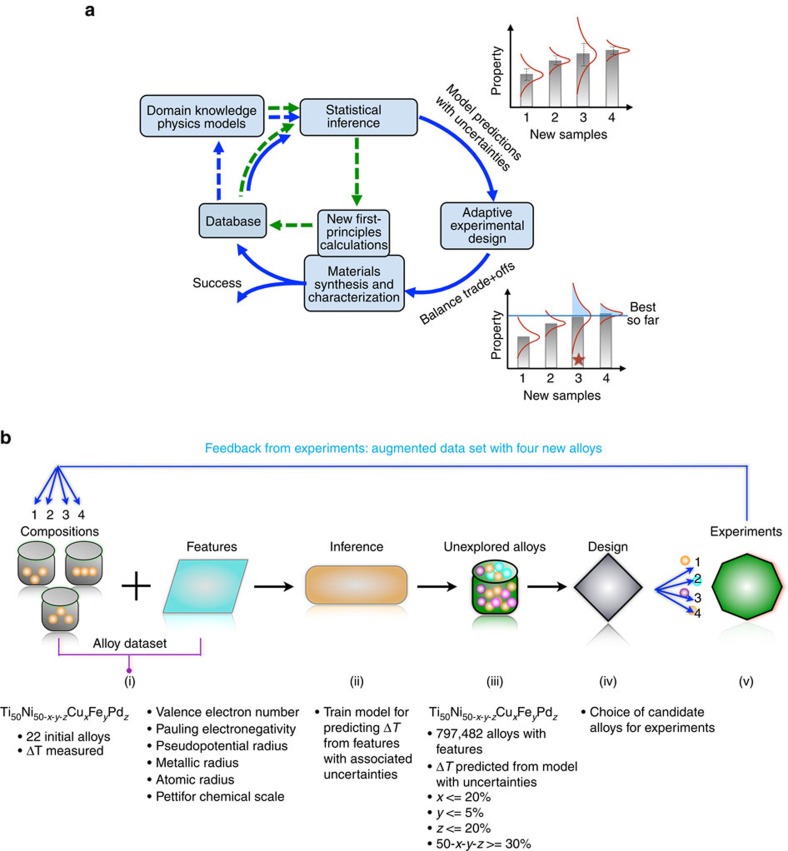
Our adaptive design loop. (**a**) Prior knowledge, including data from previous experiments and physical models, and relevant features are used to describe the materials. This information is used within a machine learning framework to make predictions that include error estimates. The results are used by an experimental design tool (for example, Global optimization) that suggests new experiments (synthesis and characterization) performed in this work, with the dual goals of model improvement and materials discovery. The results feed into a database, which provides input for the next iteration of the design loop. The green arrows represent the step-wise approach of the state-of-art using experiments or calculations, although few studies have demonstrated feedback. The red star shows that although sample number 3 is not the best predicted choice relative to sample 4, the ‘expected improvement' by selecting it is greater than other choices due to the large uncertainty. (**b**) Our loop, as executed in practice specific to the design problem featured in this work, is as follows: (i) an initial alloy experimental data set with known thermal dissipation Δ*T* and features or materials descriptors serves as input to the inference model. (ii) The model is trained and cross-validated with the initial alloy data. (iii) A data set of unexplored alloys defines the total search space of probable candidates. The trained model in (ii) is applied to all the alloys in (iii), to predict their Δ*T*. (iv) The design chooses the ‘best' four candidates for synthesis and characterization. (v) The new alloys, with their measured Δ*T*, augment the initial data set to further improve the inference and design. The four alloys for experiments are chosen iteratively by augmenting four times the initial data set with each new predicted alloy from the inference and design.

Our iterative feedback loop is schematically shown in Fig. 1a and includes the use of inference, uncertainties, global optimization and feedback from experiments (collectively referred to as ‘adaptive design'). In Fig. 1b we show how we exercise the loop, the key ingredients of which are as follows: (i) a training data set of alloys, each described by features and with a desired property (that is, Δ*T*) that has been measured; (ii) an inference model (regressor) that uses the training data to learn the feature–property relationship, with associated uncertainties; (iii) the trained model is applied to the search space of unexplored alloy compositions (for which the property has not been measured), to predict Δ*T* with associated uncertainties; (iv) design or global optimization (selector) that provides the next candidate alloy for experiment by balancing the trade-off between exploitation (choosing the material with the best predicted property) and exploration (using the predicted uncertainties to study regions of search space where the model is less accurate); and (v) feedback from experiments allowing the subsequent iterative improvement of the inference model.

### Data set

We considered Ni_50−*x*−*y*−*z*_Ti_50_Cu_*x*_Fe_*y*_Pd_*z*_ alloys with *x*, *y* and *z* compositions, where each doping element could vary in steps of 0.1% with constraints 50−*x*−*y*−*z*⩾30%, *x*⩽20%, *y*⩽5% and *z*⩽20% (see Supplementary Note 2). We synthesized 22 (training set) out of 797,504 possibilities in our group under identical conditions, to minimize the variability due to processing and microstructural effects (see Supplementary Table 1). Thus, the remaining unexplored search space consisted of 797,482 potential alloys for which Δ*T* was unknown. We measured Δ*T* using differential scanning calorimetry (DSC) and defined it as *P*_heating_−*P*_cooling_, where *P*_heating_ and *P*_cooling_ are the endothermic and exothermic peak temperatures, respectively^16^. Unlike the tangent method (an alternative approach to determine Δ*T* from DSC curves), where the estimation of transformation start and finish temperatures introduces uncertainties of the order of ±0.5 K, our approach is relatively more reliable as the DSC peaks can be determined accurately (the peak-to-peak Δ*T* has a much smaller error, <0.001 K) and our Δ*T* also correlates linearly with those from the tangent method. Additional details are present in Methods, Supplementary Figs 4 and 5, and Supplementary Note 2.

### Features

Each alloy is described in terms of one or more features, *x*, representing aspects of structure, chemistry and bonding, and the task of inference is to learn a map or model connecting *x* to Δ*T*. We used Waber–Cromer pseudopotential radii, Pauling electronegativity, metallic radius, valence electron number (VEN), Clementi's atomic radii and Pettifor chemical scale as features for the inference model^17^,^18^,^19^,^20^,^21^,^22^,^23^. There are many approaches to choosing features. We could have chosen a large number, typically of the order of 30 that could be compiled from the input, output to a density functional theory (DFT) type calculation and proceeded to perform principal component analysis to downselect a few features^5^, used methods such as gradient boosting to learn about their relative importance (not via variance) in the data^24^ or recent high-throughput approaches that distill a small number from many combinations of a given set of chosen features^6^. Our choice was dictated by prior materials knowledge. By examining a large number (60–70) NiTi-based alloys doped with Pd, Fe, Pt, Hf, Zr, Cu and so on, it has been shown^16^ that the martensitic transition temperatures (which affect thermal hysteresis) are strongly correlated with the valence electron concentration (fraction of valence electrons) and electron number per atom. In particular, the martensite and austenite start temperatures vary significantly when the valence electron concentration increases and show behaviour that depends on whether the electron valence number/atom is greater or less than 7. Moreover, the thermal hysteresis is directly influenced by the atomic size of the alloying elements (hysteresis increases with size at almost constant electron valence number)^16^. The dependence of transition temperatures and hysteresis on electron number indicates trends of variations corresponding to incomplete *d*–*d* orbital overlap at occupancy <7 and complete overlap at 7. In addition, changes in the electron number per atom influence the relative stability of various phases in the NiTiFe system^25^. In our choice of features, the Pauling electronegativity and VEN capture the chemical bonding and changes in the valence electron concentration or electron number per atom, respectively. Similarly, the Waber–Cromer pseudopotential radii, metallic radius, Clementi's atomic radii and Pettifor chemical scale were chosen to reflect the atomic size, which has been shown to influence the thermal hysteresis. Therefore, these features provide a relatively simple physical basis for predicting Δ*T* and reflect coarse-grained aspects of electronic contributions that can affect the transition temperatures and hysteresis. We potentially can reduce the number of features to 3 or 4; however, we choose to retain a higher dimensional feature space at the expense of computation. Each Ni_50−*x*−*y*−*z*_Ti_50_Cu_*x*_Fe_*y*_Pd_*z*_ alloy in our composition space was uniquely described as a weighted fraction of each of these features, where the weights are the relative concentrations (*x*, *y* and *z*) of the chemical constituents in a given alloy (see Supplementary Note 2).

### Inference

Our approach to learning from the alloy data was to establish how Δ*T* varies with the input features, *x*, by using regression methods and subsequently predict Δ*T* of unexplored alloys. Least squares or maximum likelihood are examples of such methods. However, we used several well-known regressors including a Gaussian Process Model (GPM), which is a Bayesian-based approach in terms of probability distributions, and where the means and uncertainties are natural outcomes and support vector regression (SVR), which aims to learn a nonlinear function by a mapping into a high-dimensional feature space. For the latter, we compared results using SVR with a radial basis function kernel (SVR_rbf_) and with a linear kernel (SVR_lin_), and to estimate model uncertainties we used the well-known statistical method of ‘bootstrap' sampling in which samples of the alloy data were randomly generated, allowing for replacements (Methods). These regressors produce, in general, a non-convex input/output (features/property) fitness function that may have multiple local optima. Therefore, navigating this complex fitness landscape in search of a material with optimal property solely based on regression is inadequate^7^.

### Design

To guide the next experiment, the search needs to combine exploration and exploitation, that is, explore the total experimental area and focus on a local area with the apparent global optimum. Accordingly, we employed different design functions or selectors based on a heuristic referred to as efficient global optimization (EGO) developed by Jones *et al*.^7^, which has been extensively used in the aircraft and automobile industries as part of surrogate-based optimization. We show here how this can be used for materials discovery, as this allows us to choose potential candidates for experiment based on maximizing the ‘expected improvement' over the search space. This is essentially the probability of improving the current best estimate of the target property by sampling estimates from compounds in the search space. By treating the uncertainties in their predicted values from the regressor as the realization of a normally distributed variable with mean *μ* and s.d. *σ*, the expected improvement *f*(*μ*, *σ*) can be shown to be given by *σ*[*φ*(*z*)+*z*Φ(*z*)], where *z*=(μ*−*μ*)/*σ* and μ* is the minimum Δ*T* observed in the training set, and *φ*(*z*) and Φ(*z*) are the standard normal density and distribution functions, respectively. In the limit of no uncertainty in target estimates (*σ*→0), samples will be selected with a *μ* smaller than best measured so far (μ*) (exploitation). Similarly, in the other limit (*σ*→∞), the choice will be limited to samples with the largest uncertainty (exploration) rather than seeking out those with a minimum property (exploitation). For values between the two extremes, there will be a trade-off between the two, so that local minima can be avoided, that is, the algorithm will move onto regions of greater uncertainty at the expense of lower values of Δ*T* after the local search space with potential for small Δ*T* values has been exploited. In addition to EGO, we also used the Knowledge Gradient (KG)^26^ algorithm, where the μ* is replaced by minimum over all the data, in the training and search space. We also greedily choose the next material with the best predicted value (minimum) from the regressor (pure exploitation or Min). Details are given in Methods.

### Inference and design combination

**Figure 2:**
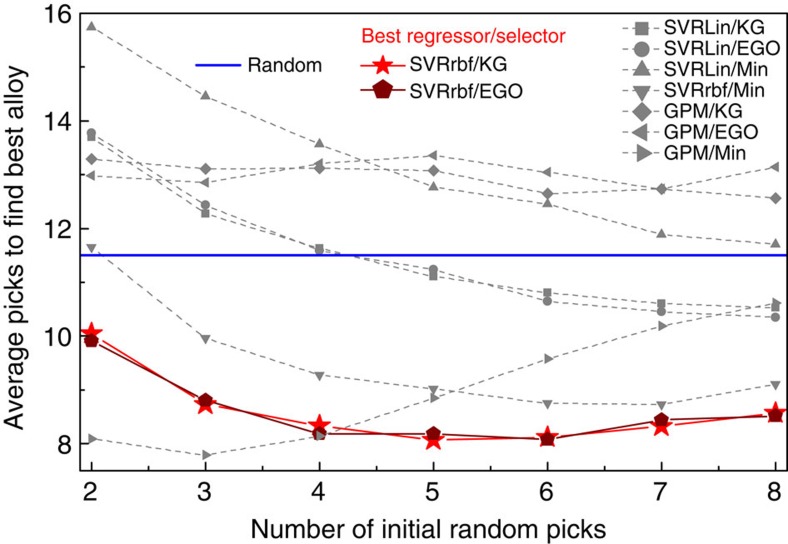
Inference and design combination. The relative performance of various regressor:selector combinations on the NiTi SMA training data set. On the abscissa, we plot the number of initial random picks, taken from the training set, for building the statistical inference model. On the ordinate, we plot the average number of picks required to find the alloy in the training set with the lowest thermal hysteresis (Δ*T*). The best regressor:selector finds the optimal alloy in as few picks as possible. We conclude that SVR_rbf_:KG (continuous red line) is the best regressor:selector combination for the NiTi SMA problem. Random picks are given as continuous blue line.

We emphasize that a priori it is not clear which regressor:selector combination to use. According to the ‘no-free-lunch theorem'^27^, there is no universal optimizer for all problems. Thus, we investigated the performances of several regressor:selector combinations as a function of the size of the data using cross-validation and found that SVR_rbf_:KG outperformed every other regressor:selector combination on the training set. Our approach (see Methods) is based on determining the average number of samples needed to find the best value when training on randomly chosen initial subsets of the training data. In particular, we randomly selected without replacement a given number of samples from the training data, trained using a given regressor:selector combination pair and counted the total number of tries needed to find the best sample in the training data. This was repeated 2,000 times with different sets of randomly selected samples and we included the initial random picks in the overall count. Figure 2 shows the relative performance of the various regressor:selector combinations on the NiTi training data. The plotting symbols are slightly larger than the s.d. of the measurements. Some of the algorithms perform worse than random, agreeing with the no-free-lunch theorem that guarantees such algorithms exist. The GPM:Min combination, which for design merely chooses the best estimate from the GPM regressor, showed the best performance for sample sizes 2 and 3; however, as more samples were included its performance began to deteriorate. For samples sizes from 4 to 8, SVR_rbf_:KG and SVR_rbf_:EGO have nearly identical performance and are better than the other regressor:selector pairs. As SVR_rbf_ works well or better than the other techniques beyond three training samples—as we have more than three training samples and as we do not have any compelling argument for using less than our full training set on the problem—the results indicate that we should choose SVR_rbf_:KG. Thus, the 22 initial samples are adequate enough for our training set, because beyond ∼5 randomly chosen training samples, we are better off using samples chosen by the design loop than by random guessing.

### New alloys via feedback from experiment

We predicted Δ*T* (using SVR_rbf_) for all the data in the search space using 1,000 ‘bootstrap' samples, to estimate *μ* and *σ* for design. As we could synthesize four compositions at a time, our predicted four compositions were obtained by incorporating the best single prediction successively in the data set, to make updated new predictions for each loop (Kriging believer algorithm^28^). Among the four samples in the first iteration, one had no martensitic transformation, whereas another gave the second best Δ*T*. The training set was augmented to 26 samples and the feedback loop of Fig. 1b repeated (see Supplementary Fig. 6 and Supplementary Note 3).

**Figure 3:**
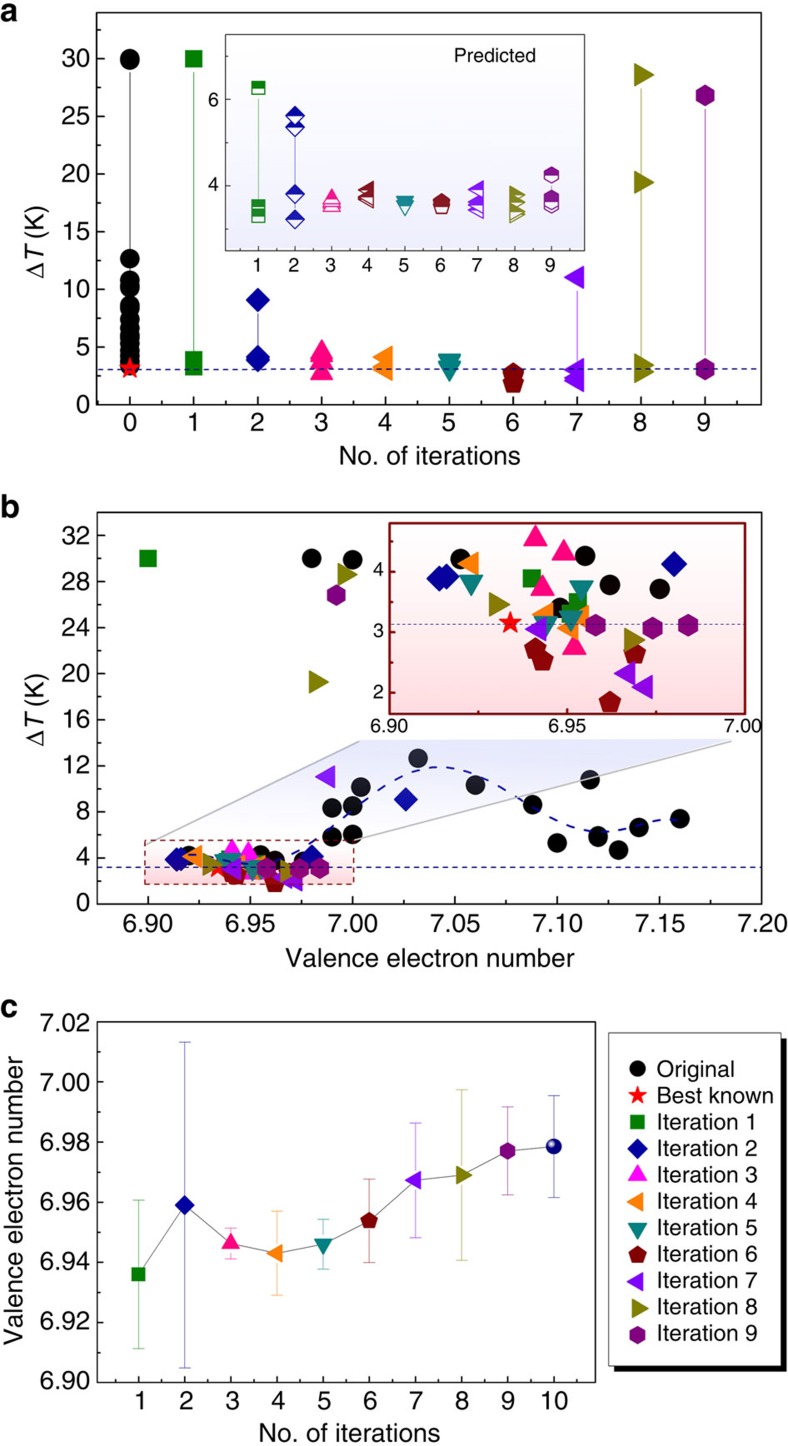
Results and insights from inference and global optimization. (**a**) The experimental measurements for thermal hysteresis Δ*T* as a function of the number of iterations of the loop of Fig. 1 compared with the predictions (inset). Iteration 0 is the original training set of 22 alloys. At each iteration (from 1 onwards), four new predicted alloys are synthesized. The difference between the predicted and measured values of Δ*T* is large for iterations 1 and 2, drops significantly for iterations 3–6 and then increases beyond iteration 7. We interpret this as illustrating exploration in the early iterations, finding a reasonable minimum in the middle iterations and then exploring new areas in later iterations. (**b**) The Δ*T* as a function of the VEN feature shows that the exploration after iteration 3 is confined to an apparent minimum in the narrow interval (6.9:7; inset), favouring the B2→R transformation that is known to have the smallest Δ*T* (global minimum) compared with B19 and B19' transformations. (**c**) The average valence electron number of the four synthesized alloys as a function of the number of iterations, showing the exploratory nature (large standard deviation (s.d.)/error bars during iterations 1–2 and from 7 onwards) of the adaptive design in this feature space. The error bars denote standard deviations for VEN over the four samples. The tenth iteration indicates that the design is drifting away from the apparent global minimum (∼6.96 in the *y* axis).

In total, we performed nine design iterations and the results are shown in Fig. 3a, which depicts how the experimental (as well as predicted, inset) Δ*T* behaves with successive iterations. The range (max–min) in Δ*T* is large in the first two iterations, becomes smaller in iterations 3–6 and increases from the seventh iteration onwards. Figure 3b shows how the measured Δ*T* varies with the average VEN (one of the features used in the inference step). There are two local minima in the VEN landscape, one at 6.95 and the other at 7.13, and SVR_rbf_:KG predominantly explores the minima for the VEN at ∼6.95, which eventually led to the discovery of the Ti_50.0_Ni_46.7_Cu_0.8_Fe_2.3_Pd_0.2_ alloy. It can be seen in Fig. 3a,b that from the third iteration onwards, our strategy produces results in the desired direction of minimizing Δ*T*. However, as shown in Fig. 3c, after the sixth iteration our design drifts away from the apparent global minimum. We found 14 new alloys, out of 36 synthesized compositions from 9 feedback loops (see Supplementary Table 2), with Δ*T* <3.15 K (the best value in our original training set). One of the alloys, Ti_50.0_Ni_46.7_Cu_0.8_Fe_2.3_Pd_0.2_ with B2 to R transformation, discovered in iteration 6 had a Δ*T* of 1.84 K (as measured from DSC curves), surpassing the best value in the training data by 42%. In Table 1, we list the best five low Δ*T* alloys from this work.

**Figure 4:**
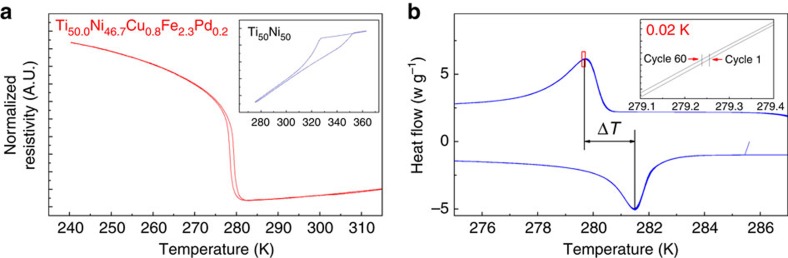
Experimental measurements for the predicted Ti_50.0_Ni_46.7_Cu_0.8_Fe_2.3_Pd_0.2_ alloy. (**a**) Resistivity measurements for the new alloy, Ti_50.0_Ni_46.7_Cu_0.8_Fe_2.3_Pd_0.2_ , compared with NiTi (inset) emphasize the very small hysteresis (0.84 K). (**b**) DSC curves for Ti_50.0_Ni_46.7_Cu_0.8_Fe_2.3_Pd_0.2_ , whose peak-to-peak Δ*T* is measured as 1.84 K, which is the lowest among related NiTi-based SMAs. Thermal cycles (60 heating and cooling cycles) also show very small shift (∼0.02 K in the inset), indicating excellent thermal fatigue resistance.

It is interesting to find that from the seventh iteration onwards, the spread in Δ*T* (see Fig. 3a) begins to widen, relative to earlier iterations. This trend could be misconstrued as arising from model overfitting. We interpret this behaviour as a consequence of our global optimization. Recall that the purpose of SVR_rbf_:KG is to balance the trade-off between exploration and exploitation. As a result, every new set of experiments are purposefully designed to rapidly learn the response surface in the high-dimensional space and minimize the model uncertainties. Therefore, Fig. 3a–c indicates that the SVR_rbf_:KG has explored the minimum in the vicinity of VEN∼6.95 and now it is moving away in search of other local minima in our response surface. We partially capture this trend in Fig. 3c, where the trend in VEN values increases continuously (it is partial, because we are in a six-dimensional feature space). Figure 4a compares the resistivity versus temperature curves (*R*(*T*)) of Ni_50_Ti_50_ and our newly found Ti_50.0_Ni_46.7_Cu_0.8_Fe_2.3_Pd_0.2_. Both *R*(*T*) curves show a reversible martensitic phase transformation but our alloy also possesses negligibly small hysteresis of 0.84 K from *R*(*T*). This thermal hysteresis is consistent with a small Δ*T* of 1.84 K measured from DSC (Fig. 4b) and the shift in transformation temperature is negligibly small, that is, ∼0.02 K (inset in Fig. 4b). In the literature, TiNiCuPd has been reported with ‘near-zero' thermal hysteresis from resistivity measurements^10^. However, for the same alloy if we use our Δ*T* yardstick, then it is determined to be 16 K. We have listed in the Supplementary Table 2 the transformation types for all the alloys we synthesized by our design loop. There are several among these, which undergo the B2 to B19 transformation. Our best B2 to B19 alloy from the design loop has a thermal hysteresis of 9.1 K as compared with 16 K for the TiNiCuPd compound^10^.

### Insights from DFT

We interpret the outcome of our inference and design in the context of energetics and strains associated with the alloy transformations. Our DFT calculations account only for the bulk or homogeneous part of the free energy and neglect interfacial, entropic or long-range elastic contributions. For small thermal dissipation across the transition, we expect the total energy difference (Δ*E*) between the austenite and martensite phases should be negative, to provide an adequate driving force for martensite transformation, and yet the magnitude (|Δ*E*|) should be relatively small, as this is a measure of the depth of the potential that has to be overcome on cooling and heating. In addition, the lattice strains associated with the phase transformations should also be small enough to allow for ease of reversibility across the transition.

Guided by Fig. 3b and our composition space, we selected three alloy families TiNiCu, TiNiFe and TiNiPd for further study. The Ti_50_Ni_34_Cu_16_ and Ti_50_Ni_46_Fe_4_ alloys with VEN values 7.16 and 6.92, respectively, fall in the two minima in Fig. 3b and Ti_50_Ni_34_Pd_16_ (VEN=7) corresponds to a data point away from the minima. Our alloy, Ti_50.0_Ni_46.7_Cu_0.8_Fe_2.3_Pd_0.2_, with the lowest Δ*T* has a VEN of 6.96, in close proximity to the Ti_50_Ni_46_Fe_4_ system. In addition, Ti_50_Ni_34_Cu_16_ and Ti_50_Ni_34_Pd_16_ both undergo a B2 to B19 transformation, whereas Ti_50_Ni_46_Fe_4_ and Ti_50.0_Ni_46.7_Cu_0.8_Fe_2.3_Pd_0.2_ display a B2 to R transformation. Our DFT calculations show that in Ti_50_Ni_34_Pd_16_, Ti_50_Ni_34_Cu_16_ and Ti_50_Ni_46_Fe_4_ alloys, Δ*E* is negative and is equal to −0.0624, −0.0337 and −0.0209, eV per atom, respectively (Supplementary Fig. 7). It is noteworthy that Δ*E* for the Ti_50_Ni_46_Fe_4_ alloy is closer to 0 (that is, its |Δ*E*| is small) relative to others. The experimental Δ*T* data for Ti_50_Ni_34_Pd_16_, Ti_50_Ni_34_Cu_16_ and Ti_50_Ni_46_Fe_4_, which are part of the training set (see Supplementary Table 1), give 8.53, 6.04 and 4.21 K, respectively, in agreement with the trend in Δ*E*.

Although Δ*E* is favourable for the R-phase, Δ*T* is also dependent on the activation barrier that must be overcome in traversing the transition. Supplementary Fig. 3 shows schematically in a Landau model context the barrier and temperature range for Δ*T*. To obtain a measure of the activation barrier, we performed DFT calculations on Ti_50_Ni_48_Fe_2_ that has Fe concentration similar to that for our Ti_50.0_Ni_46.7_Cu_0.8_Fe_2.3_Pd_0.2_ alloy and same VEN value of 6.96 (adequate Δ*T* data for Ti_50_Ni_48_Fe_2_ is not available for comparison with our alloy or Ti_50_Ni_46_Fe_4_). The objective is to obtain the energy as a function of the lattice strain (the order parameter) along a path from the austenite to the martensite for both B2 to B19 and B2 to R transformations. Experimentally, Ti_50_Ni_48_Fe_2_ undergoes a B2 to R transformation^29^ but we also considered the B2 to B19 transformation for this alloy so that the activation barriers can be compared. In these simulations we constrained all atoms in the high-symmetry position relative to the B2 phase, that is, we ‘turned off' the atomic displacements and did not relax them. We started from the high-symmetry B2 cubic structure and incrementally increased the lattice strain until we reached its maximum value (as that found in the ground-state structure) with the atoms still in the high-symmetry unrelaxed position (Supplementary Fig. 8 and Supplementary Note 4); we treated this configuration as the ‘saddle point' (although this may differ from the minimum energy path in a nudged elastic band calculation). When the atoms are also relaxed, the system then reaches its true ground state. The energy difference between the saddle point and the B2 configuration is an estimate of the activation barrier. We find that in Ti_50_Ni_48_Fe_2_ the activation barrier for B2 to R is 5.15 meV per atom, whereas that for B2 to B19 is 24.49 meV per atom, which is ∼5 times greater. Furthermore, the magnitude of the shear strain for B2 to R is less than that of the tetragonal strain for B2 to B19 (Supplementary Table 3), in agreement with the prevailing literature^30^. Thus, we conjecture that the relatively small Δ*T* in Ti_50.0_Ni_46.7_Cu_0.8_Fe_2.3_Pd_0.2_ arises from the stabilization of the R-phase due to the presence of appropriate compositions of Fe, Cu and Pd, which favour a low activation barrier and a small |Δ*E*| as well.

## Discussion

Our new alloys, even though they have very low Δ*T*, are also accompanied by relatively high transformation temperatures. The high transformation temperature was not by design and methods such as multi-objective optimization offer the potential to lead to further improvement in simultaneously optimizing both transformation temperature and Δ*T* (possibly in addition to other factors such as transformation strain, operating stress, operating temperature or processing conditions). Could our findings (14 of 36 with Δ*T* <3.15 K, the best alloy in our original set) be the result of a random occurrence? A Mann–Whitney test comparing the Δ*T* values in the original set with those in the synthesized set shows that the design loop picked alloys that are significantly better (*U*-value=172, *z* (s.d.)=3.6, *P*-value<0.001). Although the test shows that the alloys picked by the design loop are ranked better than those in the original set, we used the design loop to find the best alloys, not merely those that are better on the average. Thus, if the alloys chosen by the design loop were picked randomly from the same distribution as those in the original set, the probability that the 14 (or more) lowest Δ*T* scores would fall into the set chosen-by-the-design loop is 3.7 × 10^−4^. Clearly, the design loop is finding compounds that are better than the original alloys at significantly higher than chance performance.

## Methods

### Experimental

The base ingot of Ni_50−*x*−*y*−*z*_Ti_50_Cu_*x*_Fe_*y*_Pd_*z*_ alloy was made by arc melting of 99.9% pure Ti, 99.9% pure Ni, 99.9% pure Cu, 99.9% Fe and 99.9% pure Pd in an argon atmosphere. The ingot was then hot rolled into 1-mm-thick plate. The specimens for measurements were spark cut from the plate and then solution treated at 1,273 K for 1 h in an evacuated quartz tube, followed by water quenching. DSC measurements were made with a cooling/heating rate of 10 K min^−1^, to detect the martensitic transformation temperatures with exothermal/endothermic peaks. The desired property (Δ*T*) values were measured by using DSC with Δ*T*=*P*_heating_−*P*_cooling_, dictated by the need to have a reliable diagnostic. Additional details are discussed in Supplementary Note 2.

### Regressors

We trained regressors on samples in the training set to map features to property. The three regressors used in the present study, included the following:

- GPM
- SVR_rbf_
- SVR_lin_

The GPM is an attractive choice, because it includes an uncertainty estimate via a distribution. On the other hand, we have observed better predictive performance with other models such as SVR; however, these models do not typically estimate uncertainties. To obtain uncertainties with SVR models, we used a bootstrap approach via cross-validation. The SCIKIT-LEARN python implementations of these learning algorithms were used^31^.

### Global optimization using selectors

The selectors choose which experiment to do next by making optimal choices of materials to test. Our strategy is based on EGO^7^, to choose potential candidates by maximizing the ‘expected improvement', *f*(*μ*,*σ*), over the search space. The improvement *I* is defined by *max*(μ*−*Y*, 0), where *Y* is a random variable chosen from a distribution where the uncertainties are assumed to be normally distributed and where the mean of the property is *μ* with s.d. *σ* and μ* is the ‘best-so-far' value of the property, assuming it to be a minimum. The expected improvement, defined as *f*(*μ*,*σ*)=∫*Iφ*(*z*)*dz*, where *φ*(*z*) is the standard normal distribution, gives the improvements on the current best estimate of the target property by sampling from compounds in the search space. The integral is easily evaluated and *f* assumes the following forms for the difference selectors:

- Min: greedily choose the material in the unexplored alloy data set with minimum predicted Δ*T* value.
- EGO: maximizes the ‘expected improvement' *f*(*μ*, *σ*)=*σ*[*φ*(*z*)+*z*Φ(*z*)], where *z*=(μ*−*μ*)/*σ* and μ* is the minimum value observed so far in the training set. *φ*(*z*) and Φ(*z*) are the standard normal density and distribution functions, respectively.
- KG: *f*(*μ*,*σ*)=*σ*[*φ*(*z*)+*z*Φ(*z*)], where *z*=(μ**−*μ*)/*σ*, μ** is the minimum value of either μ* or *μ*′ and *μ*′ is the minimum predicted value in the virtual unexplored alloy data set.

In addition to these three selectors, we also employed a Random selector, which (as the name suggests) randomly selects a material without any guidance from the statistical inference model. EGO has so far been studied with GPM (Kriging), as the variance can be calculated from the distribution. However, we now use it in this work with other regressors, such as SVR_rbf_, by using ‘bootstrap sampling' to estimate the errors associated with the training set, which is considered to be representative of a sample of the overall distribution.

### Evaluation of regressor and selector combinations

As there are many ways to choose the regressor:selector pairs, we first need to choose the best regressor:selector combination. The approach we took to selecting the regressor:selector pair was to use cross-validation on the full set of training compounds we counted the average number of samples needed to find the best Δ*T* when trained on the randomly chosen subsets of the training data. For example, (a) we randomly selected (without replacement) *s*=2 samples of the training data, (b) trained using those two samples and the known Δ*T* for those samples, (c) chose the next samples from the full training set using a given regressor:selector pair and (d) counted the total number of tries needed to find the best sample in the training set. We repeated this process 2,000 times using different pairs of randomly picked samples. When counting the number of tries to find the best sample in the training set, we included the random picks in the count, for example, if we picked the best compound on the first random sample, we counted one. If we did not find the best sample in the first two random picks and had to run the regressor:selector pair three times to find the best compound, then we counted five. Therefore, every random selection of two training samples gave us a count between 1 and 22, inclusive. The average of these counts was calculated over all 2,000 cross-validation runs to obtain the average number of tries for that regressor:selector combination.

We also ran cross-validation with the same procedure but using *s*>2 random picks. We know a priori that if *s* is large, the performance will approach the (poor) performance of random picks. At the extreme, if we use *s*=21 random picks for training, all regressor:selector pairs have the same chance of finding the best compound in the first 21 random picks and all regressor:selector pairs have 100% chance of finding the best compound on the 22nd pick, if it was not found in the first 21 picks; thus, all regressor:selector combinations will perform at chance level. On the other hand, if the number of training samples is very small, for examplae, *s*=2, then we expect the regressor to perform poorly; thus, the regressor:selector combination may not perform well either. Therefore, As *s* increases we expect to see each regressor:selector performance to improve until *s* is large enough that further random picks are not as useful as regressor:selector pair to choose the next sample. This is the behaviour we see in Fig. 2.

### Density functional theory

DFT calculations for the NiTi SMAs were performed with non-spin polarized generalized gradient approximation (GGA) calculations using perdew-burke-ernzerhof (PBE) exchange-correlation functional as implemented in the QUANTUM ESPRESSO planewave pseudopotential package^32^,^33^. The core and valence electrons were treated with the normconserving pseudopotentials^34^, which were generated using OPIUM code. Solid solutions were modelled using the virtual crystal approximation^35^. We considered 60 Ry plane-wave cutoff for wavefunctions and 240 Ry kinetic energy cutoff for charge density and potential. We used the Marzari–Vanderbilt smearing^36^ with 0.02 Ry width for the Brillouin zone integration. For the Cu and Pd containing solid solutions, we performed full electronic structure calculations for B2 (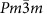, cubic) and B19 (*Pmma*, orthorhombic) phases. On the other hand, for Fe containing solid solutions, in addition to B19, we also considered the R-phase (*P*3, rhombohedral). The R-phase contains 18 atoms, which can be identified as a 3R martensitic structure and this is the structure commonly used for DFT calculations. However, experimentally the R-phase has been identified with a 9R configuration^30^. The atomic positions and the cell volume were allowed to change until an energy convergence threshold of 10^−8^ eV and Hellmann–Feynman forces <2 meV Å^−1^, respectively, were achieved. The Brillouin zone integration was performed using a 10 × 10 × 10, 10 × 8 × 8 and 4 × 4 × 6 Monkhorst–Pack *k*-point mesh^37^ centred at Γ-point for the cubic, orthorhombic and rhombohedral phases, respectively. Details and data are provided in Supplementary Note 4.

## Additional information

**How to cite this article**: Xue, D. *et al*. Accelerated search for materials with targeted properties by adaptive design. *Nat. Commun.* 7:11241 doi: 10.1038/ncomms11241 (2016).

## Supplementary Material

Supplementary Information Supplementary Figures 1-8, Supplementary Tables 1-3, Supplementary Notes 1-4 and Supplementary References

## Figures and Tables

**Table 1:** Five of the 14 best alloys with the lowest Δ*T*.

**Iterations**	**Composition**	**Δ*****T*** **(K)**	**Transformation temperature (K)**
6	Ti_50.0_Ni_46.8_Cu_0.9_Fe_2.0_Pd_0.3_	2.64	289.95
6	Ti_50.0_Ni_44.2_Cu_1.9_Fe_3.8_Pd_0.1_	2.53	243.43
6	Ti_50.0_Ni_46.7_Cu_0.8_Fe_2.3_Pd_0.2_	1.84	281.77
7	Ti_50.0_Ni_48.1_Cu_0.2_Fe_1.5_Pd_0.2_	2.09	301.86
7	Ti_50.0_Ni_46.5_Cu_1.1_Fe_2.2_Pd_0.2_	2.32	283.79

DSC, differential scanning calorimetry.

From a total of 9 iterations, which resulted in 36 new alloys, 14 had a Δ*T* <3.15 K, the lowest in the original training set of 22. Transformation temperature is given by the endothermic peak in the DSC curve.
