# Supplementary material for: Accelerated search for materials with targeted properties by adaptive design
Source: Nat Commun. 2016 Apr 15;7:11241. doi: 10.1038/ncomms11241 (PMC4835535; doi:10.1038/ncomms11241)
Supplement: Supplementary Information — Supplementary Figures 1-8, Supplementary Tables 1-3, Supplementary Notes 1-4 and Supplementary References [file ncomms11241-s1.pdf]

SUPPLEMENTARY FIGURES

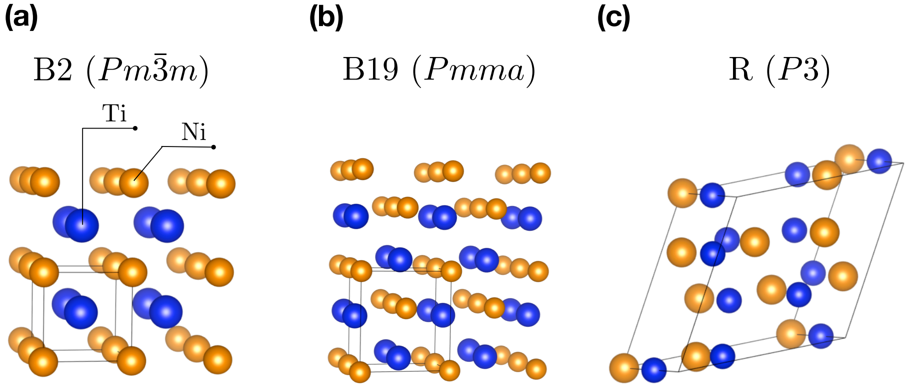

Supplementary Figure 1 | Crystal structures of the NiTi-based alloys. (a) B2, Cubic austenite structure. (b) B19, Orthorhombic martensitic structure observed in the NiTiCu and NiTiPd alloys. (c) R, rhombohedral (3R) martensitic structure observed in the NiTiFe alloys.

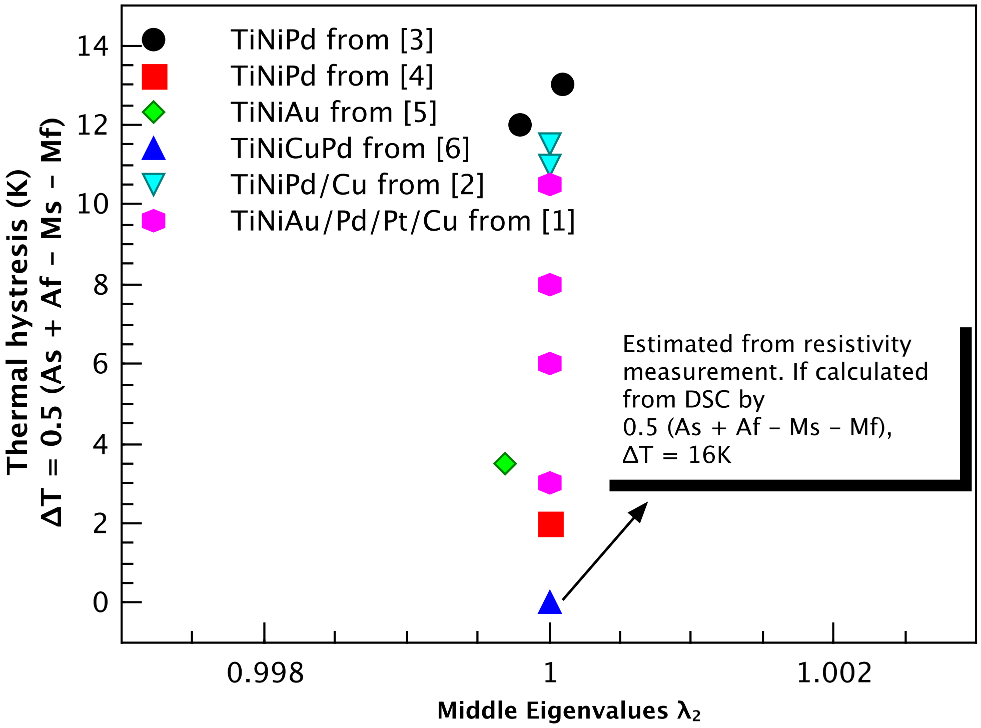

Supplementary Figure 2 | Plot of thermal hysteresis (Using $\Delta T = \frac{1}{2}(A_s + A_f - M_s - M_f)$) as a function of λ_2 in various alloys with $\lambda_2 = 1$. ΔT for the TiNiCuPd alloy was estimated from resistivity measurements. The thermal hysteresis varies from 13 K to 0 K, even though in all cases $\lambda_2 = 1$. This suggests that $\lambda_2 = 1$ is a necessary but not sufficient condition for small hysteresis.

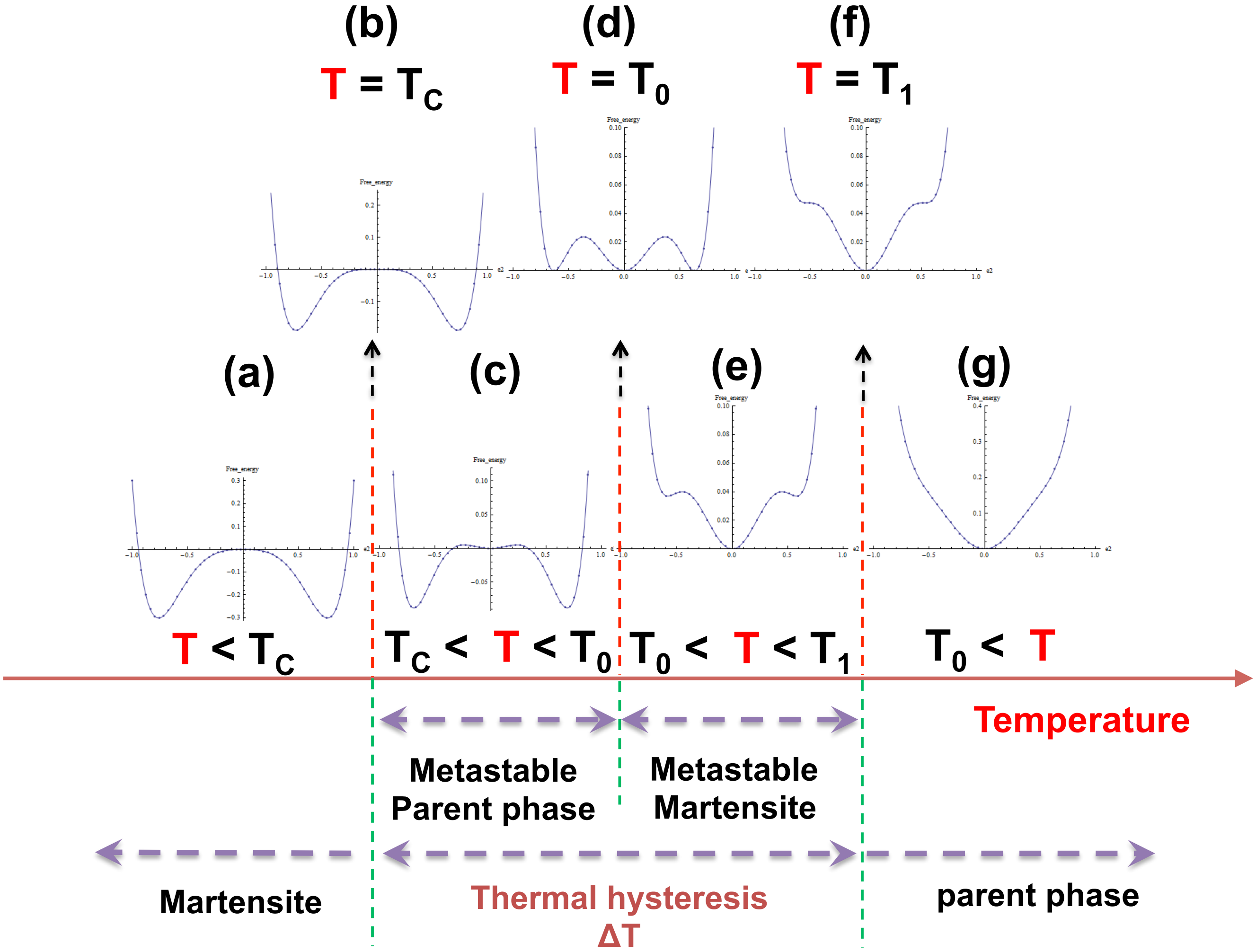

Supplementary Figure 3 | Schematic showing the evolution of the free energy landscape and its association with thermal hysteresis during heating and cooling. (a) T $< T_c$, martensite is the only stable phase; (b) T = T_c where the minimum of parent phase disappears in the free energy; (c) $T_c <$ T $< T_0$, martensite is stable and parent phase can be metastable; (d) T = T_0 where the minima of parent phase and martensite have equal free energies; (e) $T_0 <$ T $< T_1$, parent phase is stable, whereas martensite is metastable; (f) T = T_1 where the minimum of martensite disappears in the free energy; (g) T $> T_1$, parent phase is the only stable phase. During heating martensite can exist up to T_1, whereas during cooling the parent phase can exist down to T_c. The thermal hysteresis (ΔT) during heating and cooling can be considered as the temperature difference between T_1 and T_c. Such metastability is not considered by the $\lambda_2 = 1$ criterion, thus it is not a sufficient condition for discovering low thermal hysteresis alloys.

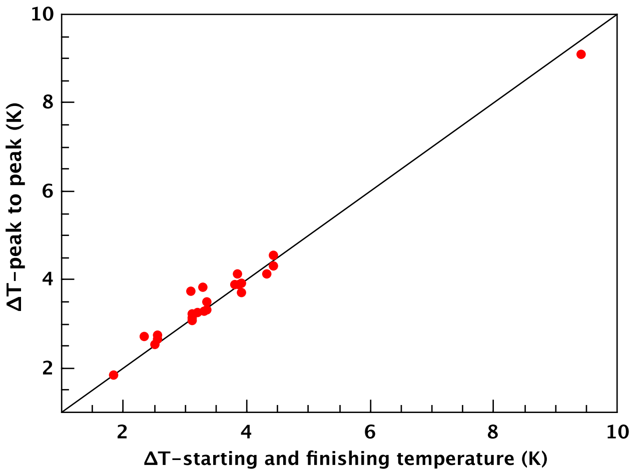

Supplementary Figure 4 | For all samples we synthesized during our design iteration loops (Table S3), $\Delta T = P_{\text{heating}} - P_{\text{cooling}}$ is linearly correlated with $\Delta T = \frac{1}{2}(A_s + A_f - M_s - M_f)$. The uncertainties in the latter are much greater than the former, as shown in Supplementary Figure 5.

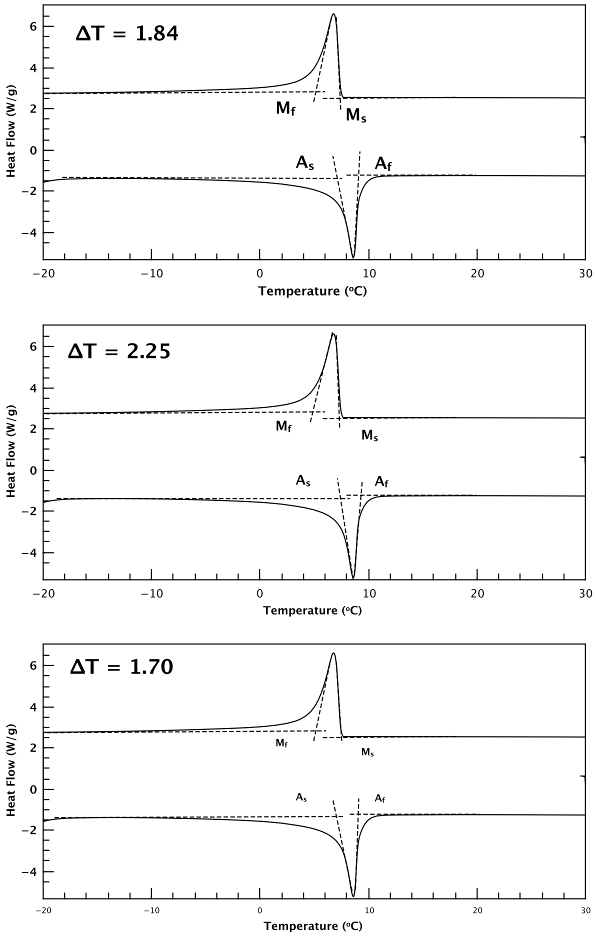

Supplementary Figure 5 | The choice of start and finish martensitic temperatures using the tangent method results in $\Delta T = \frac{1}{2}(A_s + A_f - M_s - M_f)$ varying from 1.70 K to 2.25 K. In contrast, the peak to peak method gives a ΔT of 1.84 K with an uncertainty $< .001$

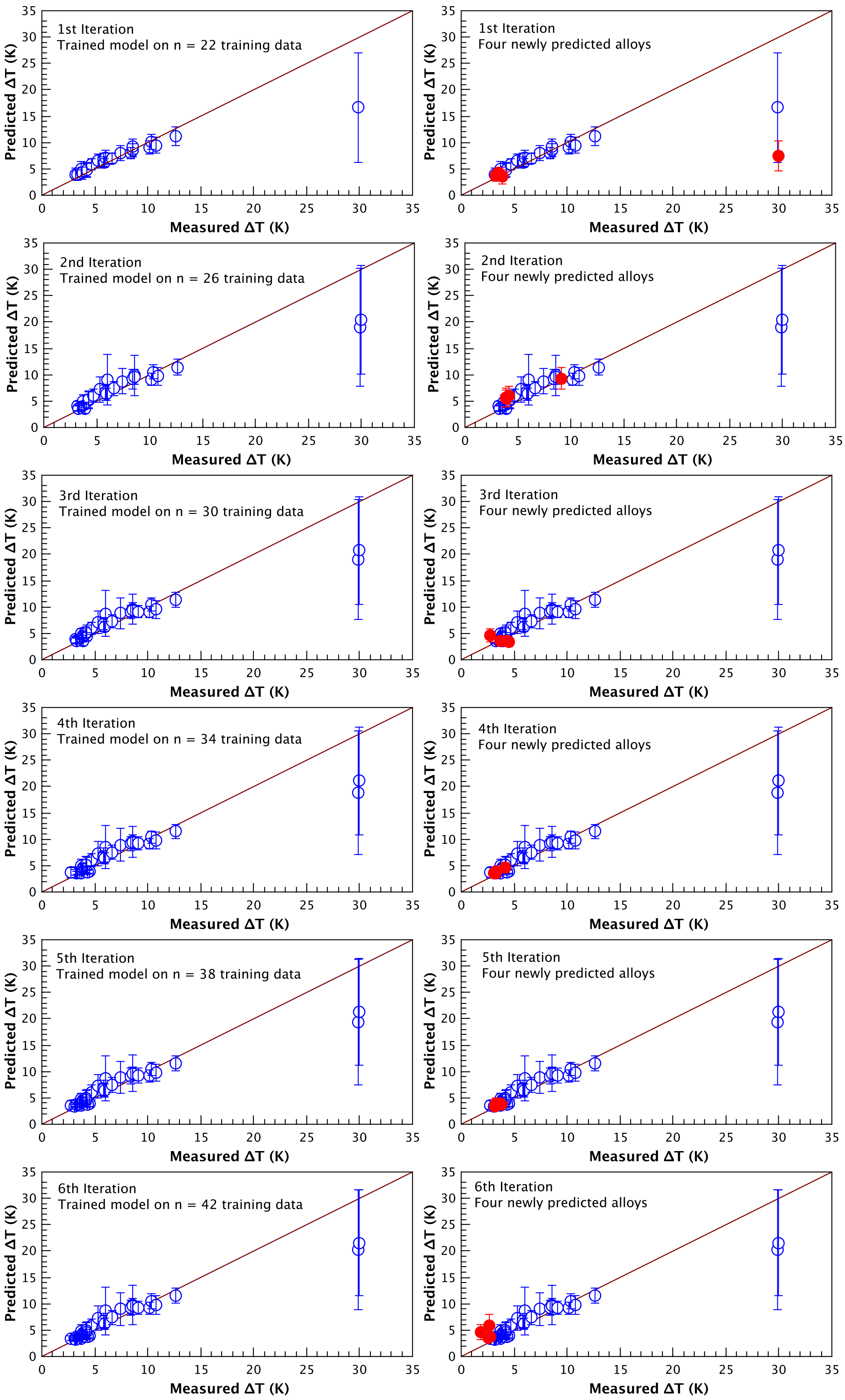

Supplementary Figure 6 | The left column shows the performance of the $\mathrm{SVR}_{\mathrm{rbf}}$ regressor at estimating ΔT at the beginning of each iteration. The blue circles show the estimated vs. actual ΔT values in the training set. The red solid points compare the predicted and experimentally measured ΔT values after each iteration.

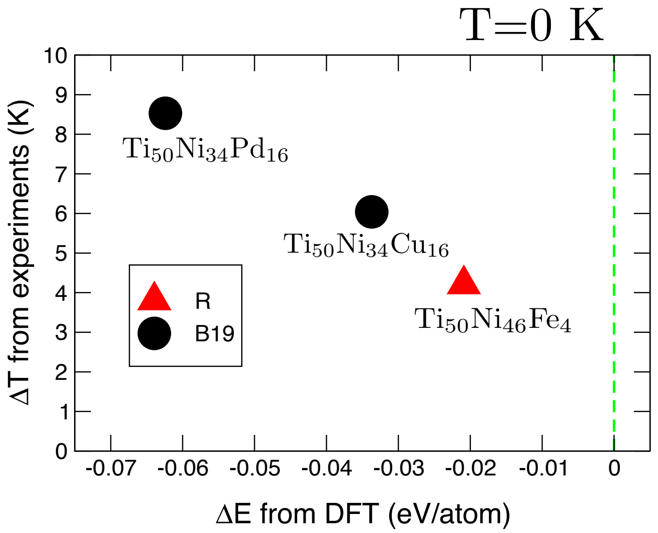

Supplementary Figure 7 | Plot of total energy difference (in eV/atom) as computed from DFT (abscissa) vs. the experimentally measured thermal hysteresis, ΔT in K, (ordinate) for $Ti_{50}Ni_{34}Pd_{16}$, $Ti_{50}Ni_{34}Cu_{16}$ and $Ti_{50}Ni_{46}Fe_4$ compositions. Negative sign indicates that the martensitic phase (B19 or R) is energetically more stable than that of the austenite phase (B2). We considered only the B19-phase for $Ti_{50}Ni_{34}Pd_{16}$ and $Ti_{50}Ni_{34}Cu_{16}$, based on recent experimental findings [1, 2]. Similarly, in the $Ti_{50}Ni_{46}Fe_4$ only R-phase has been experimentally identified [3]. ΔT values can be found in Supplementary Table 1, which is our training set for regression. ΔE is calculated as $E^{M}-E^{B2}$, where E^{M} is the total energy for the fully relaxed B19 or R structure from the virtual crystal approximation.

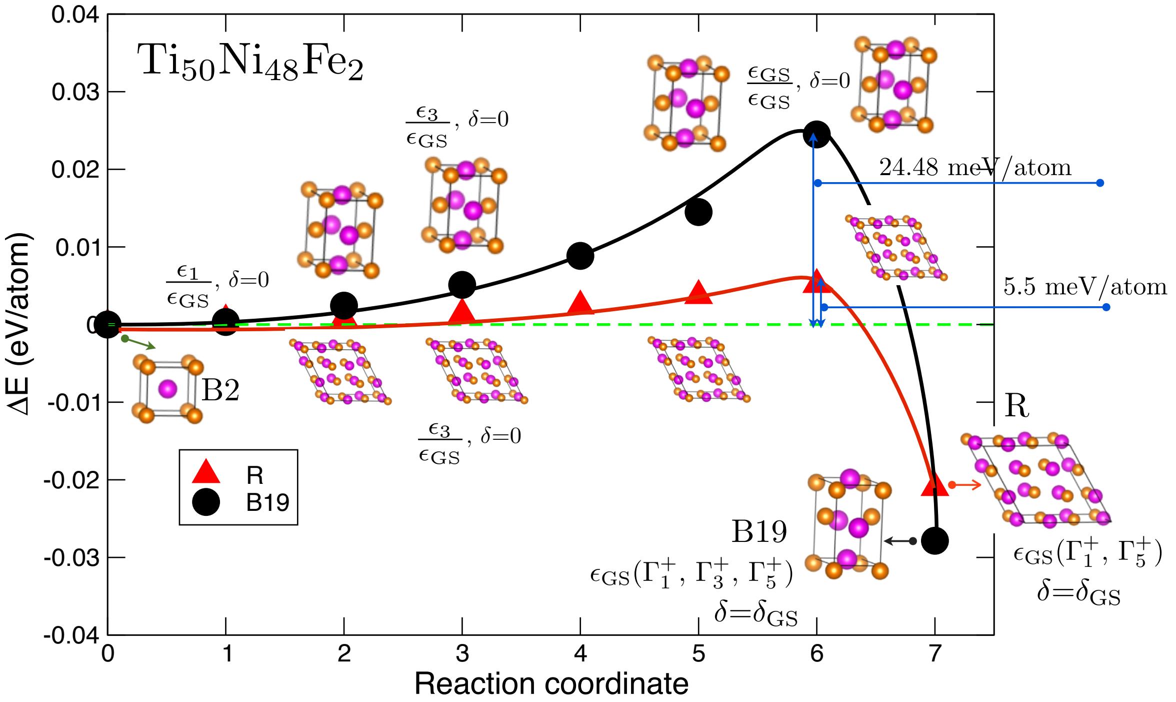

Supplementary Figure 8 | Activation barrier calculation along a reaction coordinate pathway for B2-B19 (black) and B2-R (red) phase transformation in $Ti_{50}Ni_{48}Fe_2$ alloy. Green dashed line represents the reference line that corresponds to the total energy of the B2 cubic phase. ϵ and δ indicate collective lattice strains and atomic displacements, respectively. Irreducible representations Γ_1^+, Γ_3^+ and Γ_5^+ denote the change in volume of the crystal, tetragonal strain and shear strain, respectively. In the B2-B19 and B2-R phase transformations, the order parameters are Γ_3^+ and Γ_5^+, respectively. Subscript GS stands for ground state. Reaction coordinate 0 is the high-symmetry cubic austenite phase (B2). In reaction coordinates 1 – 6, the atomic displacements (δ) were frozen and constrained to be in the unrelaxed high-symmetry positions of the B19 and R-phase. We denote this as δ=0. On the other hand, the lattice strains (order parameters) were incrementally increased from $\epsilon_1(\Gamma_1^+, \Gamma_3^+, \Gamma_5^+)$ to $\epsilon_{GS}(\Gamma_1^+, \Gamma_3^+, \Gamma_5^+)$. Activation barrier for B2-B19 and B2-R transformation is estimated as 24.48 and 5.5 meV/atom, respectively. The exact values for Γ_1^+, Γ_3^+ and Γ_5^+ strains in the ground state (ϵ_{GS}) for B19 and R structures are given in Supplementary Table 3. Reaction coordinate 7 represents the fully relaxed ground state structure.

SUPPLEMENTARY TABLES

Supplementary Table 1 | our training set with concentrations, features and properties.

Ti	Ni	Cu	Fe	Pd	cs	arc	mr	en	ven	dor	ΔT
50.00	45.20	1.00	3.80	0.00	0.9373	162.7260	135.6160	1.7219	6.9340	0.4084	3.15
50.00	44.40	2.00	3.60	0.00	0.9386	162.6720	135.6520	1.7219	6.9480	0.4082	3.40
50.00	42.80	4.00	3.20	0.00	0.9412	162.5640	135.7240	1.7220	6.9760	0.4078	3.71
50.00	43.60	3.00	3.40	0.00	0.9399	162.6180	135.6880	1.7220	6.9620	0.4080	3.78
50.00	46.00	0.00	4.00	0.00	0.9360	162.7800	135.5800	1.7218	6.9200	0.4086	4.21
50.00	44.50	1.50	3.00	1.00	0.9390	162.8500	135.7500	1.7254	6.9550	0.4104	4.26
50.00	34.00	13.00	0.00	3.00	0.9552	162.5800	136.4100	1.7324	7.1300	0.4126	4.70
50.00	34.00	10.00	0.00	6.00	0.9528	163.3000	136.6800	1.7414	7.1000	0.4202	5.32
50.00	34.00	12.00	0.00	4.00	0.9544	162.8200	136.5000	1.7354	7.1200	0.4151	5.80
50.00	42.00	5.00	3.00	0.00	0.9425	162.5100	135.7600	1.7221	6.9900	0.4076	5.83
50.00	35.00	12.00	0.00	3.00	0.9541	162.6200	136.3700	1.7325	7.1200	0.4127	5.93
50.00	40.00	0.00	0.00	10.00	0.9430	164.5000	136.8000	1.7540	7.0000	0.4312	6.04
50.00	34.00	14.00	0.00	2.00	0.9560	162.3400	136.3200	1.7294	7.1400	0.4100	6.65
50.00	34.00	16.00	0.00	0.00	0.9576	161.8600	136.1400	1.7234	7.1600	0.4049	7.40
50.00	44.00	1.00	1.00	4.00	0.9413	163.3300	136.0800	1.7357	6.9900	0.4170	8.36
50.00	34.00	0.00	0.00	16.00	0.9448	165.7000	137.5800	1.7714	7.0000	0.4457	8.53
50.00	36.40	12.00	1.60	0.00	0.9516	162.1320	136.0120	1.7225	7.0880	0.4061	8.62
50.00	41.20	6.00	2.80	0.00	0.9438	162.4560	135.7960	1.7222	7.0040	0.4073	10.16
50.00	38.00	10.00	2.00	0.00	0.9490	162.2400	135.9400	1.7224	7.0600	0.4065	10.34
50.00	34.80	14.00	1.20	0.00	0.9542	162.0240	136.0840	1.7226	7.1160	0.4057	10.79
50.00	39.60	8.00	2.40	0.00	0.9464	162.3480	135.8680	1.7223	7.0320	0.4069	12.66
50.00	50.00	0.00	0.00	0.00	0.9400	162.5000	135.5000	1.7250	7.0000	0.4070	29.89

Supplementary Table 2 | List of new NiTi-based SMAs as a function of 9 iteration cycles. No MT indicates that the chemical composition had no martensitic transformation. Data for ΔT and Transition temperature are experimentally measured. We highlight a composition (bold font), $Ti_{50.0}Ni_{46.7}Cu_{0.8}Fe_{2.3}Pd_{0.2}$, which was experimentally discovered in the 6th iteration of our adaptive design loop, with the lowest ΔT measured using the temperature difference from peak to peak in the DSC scans on cooling and heating. The nature of the transformation (final column) is based on preliminary assessment.

Iterations	Composition	Thermal hysteresis (ΔT) in K	Transformation temperature in K	Transformation type
1	$Ti_{50.0}Ni_{44.0}Cu_{2.0}Fe_{4.0}$	3.89	219.82	B2-R
1	$Ti_{50.0}Ni_{44.5}Cu_{2.1}Fe_{3.4}$	3.49	248.04	B2-R
1	$Ti_{50.0}Ni_{37.8}Fe_{5.0}Pd_{7.2}$	no MT	no MT	–
1	$Ti_{50.0}Ni_{44.0}Cu_{2.3}Fe_{3.6}Pd_{0.1}$	3.30	241.1	B2-R
2	$Ti_{50.0}Ni_{40.4}Cu_{4.6}Fe_{1.0}Pd_{4.0}$	9.10	276.48	B2-B19
2	$Ti_{50.0}Ni_{45.7}Fe_{4.3}$	3.89	230.98	B2-R
2	$Ti_{50.0}Ni_{45.8}Fe_{4.2}$	3.92	234.32	B2-R
2	$Ti_{50.0}Ni_{42.8}Cu_{3.6}Fe_{2.8}Pd_{0.8}$	4.13	234.1	B2-R
3	$Ti_{50.0}Ni_{43.9}Cu_{2.1}Fe_{4.0}$	4.54	232.81	B2-R
3	$Ti_{50.0}Ni_{44.5}Cu_{1.7}Fe_{3.7}Pd_{0.1}$	3.72	245.11	B2-R
3	$Ti_{50.0}Ni_{45.7}Cu_{1.2}Fe_{3.0}Pd_{0.1}$	2.75	264.07	B2-R
3	$Ti_{50.0}Ni_{44.0}Cu_{2.3}Fe_{3.7}$	4.31	233.79	B2-R
4	$Ti_{50.0}Ni_{45.1}Cu_{1.4}Fe_{3.5}$	3.29	247.84	B2-R
4	$Ti_{50.0}Ni_{44.6}Cu_{1.9}Fe_{3.4}Pd_{0.1}$	3.07	250.07	B2-R
4	$Ti_{50.0}Ni_{43.8}Cu_{2.6}Fe_{3.6}$	3.25	239.58	B2-R
4	$Ti_{50.0}Ni_{43.9}Cu_{1.5}Fe_{4.6}$	4.13	228.24	B2-R
5	$Ti_{50.0}Ni_{44.5}Cu_{1.9}Fe_{3.4}Pd_{0.2}$	3.15	243.1	B2-R
5	$Ti_{50.0}Ni_{46.0}Cu_{1.1}Fe_{2.8}Pd_{0.1}$	3.24	260.93	B2-R
5	$Ti_{50.0}Ni_{43.8}Cu_{2.0}Fe_{4.1}Pd_{0.1}$	3.73	230.92	B2-R
5	$Ti_{50.0}Ni_{43.9}Cu_{2.0}Fe_{4.0}Pd_{0.1}$	3.83	231.67	B2-R
6	$Ti_{50.0}Ni_{46.8}Cu_{0.9}Fe_{2.0}Pd_{0.3}$	2.64	289.95	B2-R
6	$Ti_{50.0}Ni_{44.2}Cu_{1.9}Fe_{3.8}Pd_{0.1}$	2.53	243.43	B2-R
**6**	$\mathbf{Ti_{50.0}Ni_{46.7}Cu_{0.8}Fe_{2.3}Pd_{0.2}}$	**1.84**	**281.77**	**B2-R**
6	$Ti_{50.0}Ni_{44.2}Cu_{1.9}Fe_{3.9}$	2.72	241.86	B2-R
7	$Ti_{50.0}Ni_{48.1}Cu_{0.2}Fe_{1.5}Pd_{0.2}$	2.09	301.86	B2-R
7	$Ti_{50.0}Ni_{44.5}Cu_{1.6}Fe_{3.7}Pd_{0.2}$	3.05	244.28	B2-R
7	$Ti_{50.0}Ni_{48.2}Cu_{0.6}Fe_{0.9}Pd_{0.3}$	11.05	320.34	B2-R
7	$Ti_{50.0}Ni_{46.5}Cu_{1.1}Fe_{2.2}Pd_{0.2}$	2.32	283.79	B2-R
8	$Ti_{50.0}Ni_{48.3}Fe_{1.6}Pd_{0.1}$	2.87	302.58	B2-R
8	$Ti_{50.0}Ni_{49.0}Fe_{0.2}Pd_{0.8}$	28.61	358.51	B2-B19
8	$Ti_{50.0}Ni_{48.6}Fe_{0.9}Pd_{0.5}$	19.29	332.16	B2-B19
8	$Ti_{50.0}Ni_{43.5}Cu_{2.0}Fe_{4.5}$	3.46	226.47	B2-R
9	$Ti_{50.0}Ni_{48.6}Fe_{0.8}Pd_{0.6}$	3.12	316.01	B2-R
9	$Ti_{50.0}Ni_{49.0}Fe_{0.4}Pd_{0.6}$	26.82	349.67	B2-B19
9	$Ti_{50.0}Ni_{46.1}Cu_{1.2}Fe_{2.7}$	3.12	271.81	B2-R
9	$Ti_{50.0}Ni_{48.7}Fe_{1.3}$	3.07	310.89	B2-R

Supplementary Table 3 | Amplitude (in absolute values) of the lattice strains (Γ_1^+, Γ_3^+ and Γ_5^+) in the B19 and R-phase for $Ti_{50}Ni_{48}Fe_2$ alloy whose structures are obtained from DFT calculations. Table shows the maximum lattice strain data in the fully relaxed ground state structure. `ISODISTORT` [4] program was used to obtain the lattice strain data.

Irreducible representation	B19 phase	R phase	Mode Description
Γ_1^+	0.00037	0.00027	Change in Volume
Γ_3^+	0.10178	–	Tetragonal Strain
Γ_5^+	0.06293	0.03459	Shear Strain

SUPPLEMENTARY NOTE 1

On thermal hysteresis (ΔT), compatibility (λ_2) and thermodynamics

Ni-Ti based alloys can undergo a cubic to rhombhohedral (B2 → R) or cubic to orthorhombic, monoclinic (B2 → B19, B2 → B19′) transformation as shown in Supplementary Figure 1. Although $\lambda_2 = 1$ is widely used to explain the low thermal hysteresis in shape memory alloys, it is an aspect of elastic compatibility, i.e.,$\lambda_2 = 1$ only ensures that strain compatibility is exactly satisfied between austenite and martensite. [1, 5–9] However, two or more alloys with λ_2 close to 1 can possess quite different values of thermal hysteresis, ΔT. As shown in Supplementary Figure 2, the thermal hysteresis (ΔT) varies from 13 K down to close to 0 K in different systems, even though in all cases λ_2 is very close to 1. [1, 5–9] Note that we use the definition $\Delta T = \frac{1}{2}(A_s + A_f - M_s - M_f)$ using estimates of the transition temperatures from differential scanning calorimetry (DSC) data in this plot, expect for the alloy TiNiCuPd for which ΔT is estimated from resistivity measurements. If we define ΔT as the difference in temperatures corresponding to the peak to peak height on heating and cooling, then for TiNiCuPd it is 16 K. [1] Even using data from the experimental group, the range of Δ T is from 3 K to 10 K for $\lambda_2 = 1$. [6] Therefore, $\lambda_2 = 1$ is a necessary but not sufficient condition for small hysteresis. Alloys which undergo a B2 to R transformation typically have a ΔT (measured peak to peak) in the range 3–5 K.

Thermodynamics is also an important aspect of thermal hysteresis, so that the metastability associated with first order phase transitions needs to be taken into account. As shown in Supplementary Figure 3(b), (d) and (f), there are three critical temperatures for martensitic transformation: T_c, the temperature at which the minimum of the parent phase disappears in the free energy [as shown in Supplementary Figure 3(b)]; T_0, the temperature at which the minima of both the parent and martensite phases have equal free energies [as shown in Supplementary Figure 3(d)]; and T_1, the temperature at which the minimum of the martensite phase disappears in the free energy [as shown in Supplementary Figure 3(f)]. Therefore, at a temperature below T_c [Supplementary Figure 3(a)], martensite is the stable phase, whereas at temperatures above T_0 [Supplementary Figure 3(g)] only the parent phase is stable. Metastable phases appear between T_c and T_1. For example, at temperatures above T_c, but below T_0, the stable phase is martensite but the parent phase can co-exist as a metastable phase [as shown in Supplementary Figure 3(c)]. The system has to overcome an activation barrier in traversing the transition. Similarly, at temperature above T_0 but below T_1, the stable phase is the parent phase, but martensite can be metastable [as shown in Supplementary Figure 3(d)]. Upon heating, martensite can exist up to T_1 if thermal fluctuations are not considered. Upon cooling, the parent phase expands to T_c, without considering thermal fluctuations. Therefore, the martensitic transformation does not take place at the same temperature and the thermal hysteresis (ΔT) during heating and cooling can be considered as the temperature difference between T_1 and T_c. $T_1 - T_c$ is the thermodynamic contribution to ΔT, and can be dependent upon chemistry (concentration and alloying elements). This explains why there can be large variations in ΔT for the same $\lambda_2 = 1$ condition (see Supplementary Figure 2).

SUPPLEMENTARY NOTE 2

Search Space

We constrain our problem to the $Ni_{50-x-y-z}Ti_{50}Cu_xFe_yPd_z$ family. The concentration x, y, and z are varied by step of 0.1%, and with constraints of $50\% - x - y - z \geqslant 30\%$, $x \leqslant 20\%$, $y \leqslant 5\%$ and $z \leqslant 20\%$. The size of our search space, training set, and virtual space are list below.

- search space size, N = 797504
- training set size, n = 22 out of N = 797504
- virtual set size, N - n = 797482

Note that to avoid complexity from processing conditions, raw materials, microstructure, all samples both in training set and our newly made samples were synthesized and measured in our group under the exact same conditions. Supplementary Table 1 shows our training set with concentrations, features and properties.

Thermal hysteresis (ΔT) from DSC curves

The desired property (ΔT) values were measured by using differential scanning calorimetry (DSC) with $\Delta T = P_{\text{heating}} - P_{\text{cooling}}$, dictated by the need to have a reliable diagnostic.[10] We used the temperatures for the peaks P_{heating} and P_{cooling} on heating and cooling, respectively, from the DSC scans. Typically, ΔT is obtained using the so called tangent method using $\Delta T = \frac{1}{2}(A_s + A_f - M_s - M_f)$, where M_s and M_f are the start and finish temperatures for the martensitic transformation, and A_s and A_f are the start and finish temperatures for the reverse transformation. We calculated the ΔT using both definitions for all of our newly made samples in the training set, and the results are shown in Supplementary Figure 4. The ΔT from the tangent method is linearly correlated with that obtained using peak to peak height with an $\mathfrak{R}^2$ value of 0.979. A similar dependence is obtained if we were to plot many of the other alloys from the literature. However, the uncertainitities associated with the tangent method and laboratory to laboratory variations gives a smaller $\mathfrak{R}^2$ value.

The uncertainities (± 0.5 K) in using $\Delta T = \frac{1}{2}(A_s + A_f - M_s - M_f)$ is due to the choice associated with the start and finish temperatures when employing the tangent method. As shown in Supplementary Figure 5 for our best alloy $Ti_{50.0}Ni_{46.7}Cu_{0.8}Fe_{2.3}Pd_{0.2}$, ΔT can vary from 1.70 K to 2.25 K. However, the peak to peak ΔT has a much smaller error < 0.001. We thus use P_{heating} and P_{cooling} both in our training set and for all new samples.

SUPPLEMENTARY NOTE 3

Adaptive Design Loop

1st iteration

After determining that the best regressor:selector combination for our data was SVR_{rbf}:KG using the procedure in Methods of main text, we trained the SVR_{rbf} on our n = 22 training data. We used it to predict the mean value (μ) and associated standard deviation (σ) for all samples in the virtual set by using bootstrap 1000 samples, the results are shown in Supplementary Figure 6 . We then employed KG as our selector to choose the next new compound by maximizing the expected improvement. Note that since our experimental setup allowed for the synthesis of four compositions at a time, we utilized the Kriging believer approach [11] to choose the best four candidates at a given time. This involved choosing the first alloy as before but the second choice was made after augmenting the training set with the predicted result of the first alloy, treating it as the actual measurement. This procedure was repeated for the third and fourth choices. The four selected compound were synthesized and the property (ΔT) measured using DSC. Amongst the four samples in this 1st iteration, one of them had no martensitic transformation whereas another became the alloy with the second best ΔT in the data set.

2nd to 9th iterations

The new samples become part of our training set (now n = 26), and a new regressor was trained on the data. The predicted 4 new alloys with the ΔT from the 2nd iterations are shown in Supplementary Figure 6. We repeated this procedure 9 times, and the results are shown in Supplementary Figure 6. The 36 new alloys from the 9 iterations are listed in Supplementary Table 2. One of them, $Ti_{50.0}Ni_{46.7}Cu_{0.8}Fe_{2.3}Pd_{0.2}$, has the smallest thermal hysteresis reported so far.

The predicted versus measured ΔT are shown in Supplementary Figure 6 to evaluate the performance of the regressor (until 6th iteration). We find that with successive iteration, the inference model improves in predicting the ΔT. This is clearly seen in the first and the sixth iteration for the data points whose ΔT$\sim$30 K (*i.e.* alloys that do not have martensitic transformation) and whose uncertainties are the largest.

SUPPLEMENTARY NOTE 4

Density Functional Theory

In Supplementary Figure 1, we schematically show the austenite [Supplementary Figure 1(a)] and the two martensite structures [Supplementary Figure 1(b) and (c)]. The space groups of the optimized structures from DFT were determined using `FINDSYM` [12], mode decomposition analysis was performed using `ISODISTORT` [4] and the crystal structures were visualized in `VESTA` [13].

Addition of alloying elements (e.g. Cu, Pd and Fe) to the binary $Ti_{50}Ni_{50}$ compound modifies the transformation product and/or transformation route [3]. For example, in $Ti_{50}Ni_{50}$, the phase transformation occurs between B2 cubic (austenite) and B19′ monoclinic (martensite) phases. Addition of Fe to $Ti_{50}Ni_{50}$ (where Fe occupies the Ni-site) introduces an intermediate R martensitic phase (rhombohedral) such that the $Ti_{50}Ni_{50-x}Fe_x$ alloy shows a two-stage B2-R-B19′ transformation [3]. Furthermore, at higher concentrations of Fe (x > 3%) the B19′ phase is completely suppressed [14]. Similarly, addition of Cu and Pd elements also affect the transformation product and route. $Ti_{50}Ni_{50-x}Cu_x$ alloy with 5% or more Cu shows B2-B19-B19′, where B19 is an intermediate orthorhombic martensite phase. On the other hand, Pd substitution changes the transformation to B2-B19, instead of B2-B19′. As a result, there is a complex interplay between the alloying elements, their relative concentrations, martensite product and the transformation route.

The purpose of our DFT calculation is not to establish one-to-one mapping between composition and thermal dissipation. We use inference methods (see Supplementary Figure 6) to accomplish this objective. Neither do we uncover new DFT-based descriptors that could serve as a proxy for thermal dissipation. Our goal is to glean insights into the low ΔT behavior of $Ti_{50.0}Ni_{46.7}Cu_{0.8}Fe_{2.3}Pd_{0.2}$ alloy and to do so, we perform two types of DFT calculations:

1. We choose alloys whose ΔT values and martensite phases are known from experiments and have different valence electron number (VEN) values. For these alloys, we compute the total energies in both the austenite (B2) and martensite phases (R or B19, whichever is experimentally observed). We then calculate the total energy difference, ΔE (where $\Delta E = E^{martensite} - E^{B2}$). For small thermal dissipation, it is reasonable to assume that an alloy should have negative ΔE that provides an adequate driving force for martensite transformation and yet the magnitude ($|\Delta E|$) should be relatively small as this is a measure of the depth of the potential that has to be overcome on cooling and heating.

2. In addition to having a favorable $|\Delta E|$, the activation barrier should also be small for low ΔT. This is schematically shown in Supplementary Figure 2. We calculate the activation barrier by choosing a TiNiFe alloy whose Fe-concentration is more or less similar to the newly discovered $Ti_{50.0}Ni_{46.7}Cu_{0.8}Fe_{2.3}Pd_{0.2}$ alloy and has the same VEN. It is strictly not necessary to know the experimental ΔT value for this TiNiFe alloy (unlike the previous case in Bullet 1, where the knowledge of ΔT is critical). We estimate its activation barriers for B2-R and B2-B19 along a path dictated by the lattice strain (order parameter here) and compare them to determine the energetically favorable transformation product.

In Supplementary Figure 7, the total energy differences (ΔE) from DFT between the austenite and martensite phases for the $Ti_{50}Ni_{34}Pd_{16}$, $Ti_{50}Ni_{34}Cu_{16}$ and $Ti_{50}Ni_{46}Fe_4$ compositions are shown. As noted in the main manuscript and Bullet 1 above, the $Ti_{50}Ni_{34}Cu_{16}$ and $Ti_{50}Ni_{46}Fe_4$ alloys with valence electron number (VEN) values 7.16 and 6.92, respectively, fall in the two minima in Figure 3b (in the main manuscript) and $Ti_{50}Ni_{34}Pd_{16}$ (VEN=7) corresponds to a data point away from the minima. We considered only the B19-phase for $Ti_{50}Ni_{34}Pd_{16}$ and $Ti_{50}Ni_{34}Cu_{16}$, based on recent experimental findings [1, 2]. Similarly, in the $Ti_{50}Ni_{46}Fe_4$ only R-phase has been experimentally identified [3]. Experimental ΔT for these alloys in their respective martensite phases can be found in the Supplementary Table 1. It should be noted that our DFT calculations use the virtual crystal approximation (VCA), where we average the pseudopotentials of the two atoms that have partial site occupancy and do not consider the local effects. As a result, the outcome and energy trends from our DFT calculations are at best qualitative.

In addition to the three alloys given in Supplementary Figure 7, we also performed DFT calculations on $Ti_{50}Ni_{48}Fe_2$ to accomplish the objective stated in Bullet 2. Note that $Ti_{50}Ni_{48}Fe_2$ has Fe-concentration similar to that of our $Ti_{50.0}Ni_{46.7}Cu_{0.8}Fe_{2.3}Pd_{0.2}$ alloy and same VEN value of 6.96. However, adequate ΔT data for $Ti_{50}Ni_{48}Fe_2$ from our DSC measurement is not available for comparison. The goal is to determine the activation barrier (Supplementary Figure 3) for B2-B19 and B2-R transformation in the $Ti_{50}Ni_{48}Fe_2$ alloy and gain insights on the low ΔT behavior of $Ti_{50.0}Ni_{46.7}Cu_{0.8}Fe_{2.3}Pd_{0.2}$ alloy. It is well known that the crystallography of the martensitic phase (whether it is B19 or R) has a key effect on the hysteresis. Generally, the R-phase has a smaller dissipation compared to the B19-phase, due to the smaller transformation strain involved during the austenite to martensite phase transformation [3].

For $Ti_{50}Ni_{48}Fe_2$, we fully relaxed the lattice and atomic positions in both B19 and R structures. In Supplementary Table 3, the relative amplitudes for the lattice strain modes (order parameters) for the $Ti_{50}Ni_{48}Fe_2$ alloy in B19 and R structures are given. Clearly, the tetragonal strain (Γ_3^+) and shear strain (Γ_5^+) manifests as the order parameters for B19- and R-phases, respectively. Furthermore, the amplitude of Γ_5^+ for B2-R is less than that of the Γ_3^+ for B2-B19.

We then performed a series of computational simulations to estimate the activation barrier for $Ti_{50}Ni_{48}Fe_2$ in the B2-B19 and B2-R phase transformation. The results are given in Supplementary Figure 8. Our calculations show that the activation barrier for B2-R is $\sim$5 times smaller than that for the B2-B19 transformation. For completeness, the barrier for B2-B19$'$ is also estimated to be 67.01 meV/atom, which is much higher than that for the B2-B19 transformation (data not shown in Supplementary Figure 8). Ideally, a variable-cell nudged elastic band calculation [15] would provide a more accurate estimation of the activation barrier; however this method is not implemented in the `Quantum ESPRESSO` package [16] used in this work.

SUPPLEMENTARY REFERENCES

[1] Robert Zarnetta, Ryota Takahashi, Marcus L. Young, Alan Savan, Yasubumi Furuya, Sigurd Thienhaus, Burkhard Maa, Mustafa Rahim, Jan Frenzel, Hayo Brunken, Yong S. Chu, Vijay Srivastava, Richard D. James, Ichiro Takeuchi, Gunther Eggeler, and Alfred Ludwig, Identification of Quaternary Shape Memory Alloys with Near-Zero Thermal Hysteresis and Unprecedented Functional Stability, Advanced Functional Materials **20**, 1917–1923 (2010).

[2] X.L. Meng, H. Li, W. Cai, S.J. Hao, and L.S. Cui, Thermal cycling stability mechanism of $Ti_{50.5}Ni_{33.5}Cu_{11.5}Pd_{4.5}$ shape memory alloy with near-zero hysteresis, Scripta Materialia **103**, 30 – 33 (2015).

[3] K. Otsuka and X. Ren, Physical metallurgy of Ti–Ni-based shape memory alloys, Progress in Materials Science **50**, 511 – 678 (2005).

[4] Branton J. Campbell, Harold T. Stokes, David E. Tanner, and Dorian M. Hatch, ISODISPLACE: a web-based tool for exploring structural distortions, Journal of Applied Crystallography **39**, 607–614 (2006).

[5] Jun Cui, Yong S Chu, Olugbenga O Famodu, Yasubumi Furuya, Jae Hattrick-Simpers, Richard D James, Alfred Ludwig, Sigurd Thienhaus, Manfred Wuttig, Zhiyong Zhang, *et al.*, Combinatorial search of thermoelastic shape-memory alloys with extremely small hysteresis width, Nature materials **5**, 286–290 (2006).

[6] Zhiyong Zhang, Richard D James, and Stefan Müller, Energy barriers and hysteresis in martensitic phase transformations, Acta Materialia **57**, 4332–4352 (2009).

[7] Remi Delville, Sakthivel Kasinathan, Zhiyong Zhang, Jan Van Humbeeck, Richard D James, and Dominique Schryvers, Transmission electron microscopy study of phase compatibility in low hysteresis shape memory alloys, Philosophical magazine **90**, 177–195 (2010).

[8] Xian Chen, Vijay Srivastava, Vivekanand Dabade, and Richard D James, Study of the cofactor conditions: Conditions of supercompatibility between phases, Journal of the Mechanics and Physics of Solids **61**, 2566–2587 (2013).

[9] H Shi, R Delville, V Srivastava, RD James, and D Schryvers, Microstructural dependence on middle eigenvalue in Ti–Ni–Au, Journal of Alloys and Compounds **582**, 703–707 (2014).

[10] Mehrdad Zarinejad and Yong Liu, Dependence of transformation temperatures of NiTi-based shape-memory alloys on the number and concentration of valence electrons, Advanced Functional Materials **18**, 2789–2794 (2008).

[11] Noel Cressie, The origins of kriging, Mathematical Geology **22**, 239–252 (1990).

[12] Harold T. Stokes and Dorian M. Hatch, FINDSYM: program for identifying the space-group symmetry of a crystal, Journal of Applied Crystallography **38**, 237–238 (2005).

[13] Koichi Momma and Fujio Izumi, *VESTA*: a three-dimensional visualization system for electronic and structural analysis, Journal of Applied Crystallography **41**, 653–658 (2008).

[14] Dong Wang, Zhen Zhang, Jian Zhang, Yumei Zhou, Yu Wang, Xiangdong Ding, Yunzhi Wang, and Xiaobing Ren, Strain glass in Fe-doped Ti–Ni, Acta Materialia **58**, 6206–6215 (2010).

[15] Guang-Rui Qian, Xiao Dong, Xiang-Feng Zhou, Yongjun Tian, Artem R. Oganov, and Hui-Tian Wang, Variable cell nudged elastic band method for studying solid–solid structural phase transitions, Computer Physics Communications **184**, 2111 – 2118 (2013).

[16] Paolo Giannozzi, Stefano Baroni, Nicola Bonini, Matteo Calandra, Roberto Car, Carlo Cavazzoni, Davide Ceresoli, Guido L Chiarotti, Matteo Cococcioni, Ismaila Dabo, Andrea Dal Corso, Stefano de Gironcoli, Stefano Fabris, Guido Fratesi, Ralph Gebauer, Uwe Gerstmann, Christos Gougoussis, Anton Kokalj, Michele Lazzeri, Layla Martin-Samos, Nicola Marzari, Francesco Mauri, Riccardo Mazzarello, Stefano Paolini, Alfredo Pasquarello, Lorenzo Paulatto, Carlo Sbraccia, Sandro Scandolo, Gabriele Sclauzero, Ari P Seitsonen, Alexander Smogunov, Paolo Umari, and Renata M Wentzcovitch, QUANTUM ESPRESSO: a modular and open-source software project for quantum simulations of materials, Journal of Physics: Condensed Matter **21**, 395502 (19pp) (2009).
